# The Way to Educate Medical Students

**Published:** 1890-04

**Authors:** 


THE WAY TO EDUCATE MEDICAL STUDENTS.—The continuous agitation by medical journals of the subject of the faults of medical education in this country has begun to bear some fruit. It is among the most significant results that the public begins to show a little interest in the matter. The daily press often treates of medical education editorially, and critical reviews publish articles about it. The *North American Review* has recently contained an article entitled "The Open Door of Quackery," written by three physicians well known as educators, Drs. Austin Flint and Eggleston, and Professor Doremus. In this article some plain statements are made regarding the deficiencies of American medical colleges. Dr. Eggleston, for example, says—and with perfect truth—"that there are not a dozen American medical colleges out of the one hundred and seventeen that would be tolerated for a moment in any foreign country that pretends to be civilized."

When the public generally knows that the above statement is correct, more care will be taken in selecting the family physician; and after a time it will, we trust, come about that intelligent people will not patronize medical men who have not had a thorough educational training, any more than they would hire an engineer who had not been carefully prepared for his profession, to build a difficult railroad, or to bridge a dangerous pass.

The two chief deficiencies in medical colleges just now are lack of the requirement of a good preliminary education and lack of facilities for clinical teaching and for technical instruction in the special branches. The latter requisite is being supplied measurably by post-graduate schools, and by the multiplication of small hospitals. The question of preliminary education is, however, often a serious one.

The physician needs to be not only a medical man with technical skill, but a man of some general culture and worldly knowledge. These latter elements are almost as important as the former. The worst way to make a physician is to take a youth of sixteen, put him at once into exclusively scientific studies, follow these up with medicine in all its branches, hospitals, laboratories, clinics, until by the time he is twenty-three he is a monument of technical knowledge. Such a course makes good operators in certain kinds of work; it produces a narrow specialistic skill, but not good general practitioners.

Preparatory schools of medicine are, we sincerely believe, useless and pernicious institutions.

The best preliminary training is undoubtedly such as is now given in our smaller colleges, but so modified in the last two years that the graduate in arts can graduate in medicine after two years of study in a medical college having nine months terms. Then, with a year or two spent in hospitals or post-graduate courses, the student is ready to do good work in his profession, and he need not be more than twenty-four or five years of age.—*Editorial in Medical Record.*

